# Population-Level Uncoupling of Antimicrobial Usage and Resistance in Community-Onset *Escherichia coli* Bloodstream Infections

**DOI:** 10.3390/pathogens15070670

**Published:** 2026-06-25

**Authors:** Peter Collignon, John J. Beggs, Jan M. Bell, Denise Daley, Elizabeth Roughead, on behalf of the Australian Group on Antimicrobial Resistance

**Affiliations:** 1 Medical School, Australian National University, Canberra 2601, Australia; 2 Microbiology, Canberra Hospital, Canberra 2605, Australia; 3 Independent Researcher, Melbourne 3000, Australia; 4 Australian Group on Antimicrobial Resistance (AGAR), Perth 6000, Australiadenise.daley@health.wa.gov.au (D.D.); 5 Microbiology, Fiona Stanley Fremantle Hospitals Group, Murdoch 6961, Australia; 6 College of Health, Adelaide University, Adelaide 5000, Australia; libby.roughead@adelaide.edu.au

**Keywords:** antibiotics, antimicrobial resistance, AMR, age, sex, *E. coli*, gender, sex: elderly, bloodstream infections

## Abstract

Background: Antimicrobial resistance (AMR) is widely considered to be driven by antimicrobial consumption through within-host selection. However, whether this mechanism adequately explains population-level patterns of resistance in invasive infections remains uncertain. If antimicrobial use is the dominant determinant, resistance should be highest in demographic groups with the greatest exposure. Methods: We conducted a retrospective analysis of 44,792 community-onset *Escherichia coli* bloodstream infection episodes identified through national Australian surveillance data (2013–2024). Resistance prevalence across individual antimicrobials and composite multidrug resistance panels was analysed by age and sex. These data were compared with community antimicrobial dispensing derived from the Pharmaceutical Benefits Scheme. Mean resistance was modelled as a function of age and sex. Results: Antimicrobial use was substantially higher in females than males (~23% overall) and increased markedly with age, with individuals aged ≥80 years receiving approximately three times more antimicrobials than those aged 25–30 years. In contrast, resistance was consistently lower in females across most antimicrobials and composite measures. Resistance demonstrated an inverted U-shaped age distribution, peaking at 30–40 years before declining in older age groups. From early adulthood to older age, antimicrobial dispensing increased threefold, whereas mean resistance declined by approximately 20%. These patterns were consistent across antimicrobial classes, years, and jurisdictions. Conclusions: These findings show that demographic patterns of antimicrobial resistance in community-onset *E. coli* bloodstream infections are not well explained by a simple population-level consumption model. These findings should be interpreted as important hypothesis-generating insights. Although antimicrobial exposure remains important for individual-level selection, the observed discordance between prescribing and resistance suggests that other factors, including differences in transmission pathways, healthcare contact, disease prevalence, community sanitation and socioeconomic circumstances may also significantly shape resistance patterns.

## 1. Introduction

Antimicrobial resistance (AMR) is a major global health threat, contributing substantially to morbidity, mortality, and healthcare burden worldwide [1,2,3]. The prevailing paradigm holds that antimicrobial consumption is the principal driver of resistance, based on well-established within-host selection: exposure to antibiotics increases the probability that resistant organisms emerge and persist [3,4,5]. This framework underpins most antimicrobial stewardship strategies, which aim to reduce resistance through reductions in antimicrobial use.

However, the extent to which antimicrobial consumption alone explains population-level patterns of resistance, particularly in serious invasive infections, remains uncertain. At ecological scales, associations between antimicrobial use and resistance are often heterogeneous across settings, time periods, and pathogens [6,7,8,9,10]. Increasingly, evidence suggests that transmission dynamics—including environmental exposure from contaminated water, food systems where multi-resistant bacteria are frequently present, poor sanitation that allows multi-resistant bacteria to reach drinking water or water for crops and vegetables, healthcare contact, as hospitals frequently have higher levels of resistant bacteria than the community, and travel to regions with high levels of multi-resistant bacteria in the community—all can play a substantial role in shaping the distribution of antimicrobial resistance [6,7,8,9,10,11,12,13].

These considerations are particularly relevant for *E. coli*, a leading cause of bloodstream infection globally. Unlike pathogens primarily transmitted within healthcare settings, *E. coli* circulates across multiple reservoirs, including humans, animals, food, water, and the environment [3,6,7,8,9,10,11,12,13]. In this context, antimicrobial resistance may reflect not only within-host selection but also the acquisition and spread of resistant strains through complex transmission pathways; all aspects of a One Health approach to better manage AMR [12,13].

Age and sex are fundamental determinants of antimicrobial exposure. In many settings, females receive more antimicrobials than males, and antimicrobial use increases substantially with age [14,15,16,17,18,19,20,21,22,23,24]. If antimicrobial consumption were the dominant determinant of resistance at the population level, resistance would be expected to be highest in these groups. However, few studies have examined antimicrobial use and resistance jointly across both age and sex in large datasets of clinically significant infections. We in this study are looking at all those factors together. Moreover, major surveillance systems, including GLASS, do not routinely disaggregate resistance and usage data by these variables [25].

In this study, we analyse national Australian data on 44,792 episodes of community-onset *E. coli* bloodstream infection over a 12-year period. By examining antimicrobial resistance alongside antimicrobial use across age and sex strata, we examine whether observed demographic patterns are consistent with a simple consumption-driven model of resistance, and which presumes that nearly all levels of resistance seen are dependent on antibiotic consumption. We further consider whether alternative explanations, including differences in exposure and transmission, may better account for the observed patterns.

## 2. Methods

### 2.1. Study Design

Antimicrobial susceptibility data were sourced from up to 32 laboratories servicing 57 hospitals across Australia. Each laboratory collected either all, or up to 200 isolates, from different patient episodes of bloodstream infections per year, between 1 January 2013 and 31 December 2024 of *Enterobacterales*, *Acinetobacter* species, or *Pseudomonas aeruginosa*. In patients with more than one isolate, a new episode was defined as a new positive blood culture if collected more than two weeks after the initial positive culture [26,27]. An episode was defined as community-onset if the first positive blood culture was collected 48 h or less after admission. The size, geographical breadth and time span of the antimicrobial susceptibility data provide protection against biases that might arise from episodic location specific and time specific conditions.

### 2.2. Species Identification

Isolates were identified using the routine method at each institution which included either the Vitek^®^ 2 (bioMérieux, Lyon, France) or the Phoenix™ (Becton Dickinson, Franklin Lakes, NJ, United States of America) automated microbiology systems, or matrix assisted laser desorption/ionisation-time of flight (MALDI-TOF) mass spectrometry.

### 2.3. Antimicrobial Susceptibility Testing

Antimicrobial susceptibility tests (AST) were performed using either the Vitek^®^ 2 or Phoenix™ commercial automated susceptibility methods which were calibrated to the ISO reference standard method of broth microdilution. Various commercially available Vitek^®^ 2 cards (AST-N246, AST-N434, AST-N435, AST-N410) or the Phoenix NMIC-422 card were utilized throughout the survey period, according to routine standard antimicrobial testing protocol of each participating laboratory for each year. The European Committee on Antimicrobial Susceptibility Testing (EUCAST) version 15.0 breakpoints from January 2025 were employed in the analysis [26,27]. We did this so we are using consistent MIC breakpoint criteria over all the years to assess resistance.

### 2.4. Antimicrobials Studied

Although results from 25 antimicrobials were available, six antimicrobials (aztreonam, cefuroxime, ertapenem, fosfomycin, imipenem, tigecycline) were excluded from the study because they were tested for less than 10% of the total isolates. In addition, amoxycillin-clavulanic acid results were not analysed as the formulation of amoxicillin–clavulanic acid in some of the Vitek^®^ cards prevented application of EUCAST interpretive guidelines. Furthermore, only antimicrobials suitable for systemic use were analysed. Thus cefalexin, mecillinam, nitrofurantoin, norfloxacin, and trimethoprim results were excluded as the EUCAST breakpoints only apply for uncomplicated urinary tract infections. Antimicrobials included in the study are summarised in Supplementary Table S1. Supplementary Table S2 summarises the sample size by age category and sex.

### 2.5. Analysis

To compare antimicrobial resistance rates to community antimicrobial usage, analyses were restricted to bloodstream infection episodes with a community-onset, i.e., blood culture collected 48 h or less after hospital admission.

We assessed three antimicrobial panels: a 12-drug panel, a 6-drug panel and a 5-drug panel.

The 12-drug panel consisted of those drugs for which a result was available from 2013 to 2024, and included, ampicillin, piperacillin and tazobactam, cefazolin, ceftriaxone, ceftazidime, cefepime, meropenem, ciprofloxacin, gentamicin, tobramycin, amikacin, and trimethoprim/sulfamethoxazole.

The 6-drug panel consisted of a representative drug for each major systemic drug class and included ampicillin, ceftriaxone, meropenem, ciprofloxacin, gentamicin, and trimethoprim/sulfamethoxazole.

The 5-drug panel excluded ampicillin from the 6-drug panel. Ampicillin was excluded as ampicillin resistance was generally greater than 50%, which had the potential to distort the data.

Overall *E. coli* antimicrobial resistance of each individual was measured as the number of drugs in each panel to which that person’s infection was resistant. Mean resistance was defined as the arithmetic average of the number of resistant drugs in a panel of persons in each sex and age defined demographic group.

Descriptive statistics were employed to report antimicrobial usage rates per capita and antimicrobial resistance by age and sex and calendar years calculated as rates per person both for individual antimicrobials and multidrug panels of antimicrobials. Mean drug resistance was analysed at the episode level using ordinary least squares regression. The dependent variable was the number of antimicrobials to which each isolate was resistant within the relevant panel. Separate models were fitted for each antimicrobial panel and sex, with age and age squared included as continuous covariates to allow for non-linear age effects. Fitted values from these models were overlaid on age-band descriptive plots. Model coefficients, standard errors, *p*-values, R^2^ values, residual standard errors, and estimated peak ages were reported in the Supplementary Tables. These models were used descriptively to summarise age-related patterns, not to infer individual causal effects of antimicrobial exposure.

### 2.6. Antimicrobial Usage Data

Antimicrobial usage, as measured by dispensing supplied under the Australian Pharmaceutical Benefits Scheme (PBS) was available by 5-year age ranges and sex from the publicly available Antimicrobial Use and Resistance Australia (AURA) report 2024 [24]. The data included antibiotics dispensed to all Australians and permanent residents eligible for Medicare representing a whole-of-population health care system. The PBS dataset does not include in-patient supply within public hospitals and thus represents predominantly community usage. To confirm usage by sex was consistent over time, data from the 10% sample of the Pharmaceutical Benefits were analysed using the same methods employed for AURA. The antimicrobials included are listed in Supplementary Table S1. The analysis of the 10% sample was approved by the External Requests Evaluation Committee RMS4567.

Antimicrobial usage in the community for 2013–2024 was used for comparison against antimicrobial resistance over the same time period [28,29]. Antimicrobial prescriptions per capita of resident population per year by age category and sex, and the ratio of female to male antimicrobial prescriptions per capita of resident population per year by age category and sex are summarised in Supplementary Tables S3 and S4.

## 3. Results

### 3.1. Antimicrobial Usage by Age and Sex

**Figure 1:**
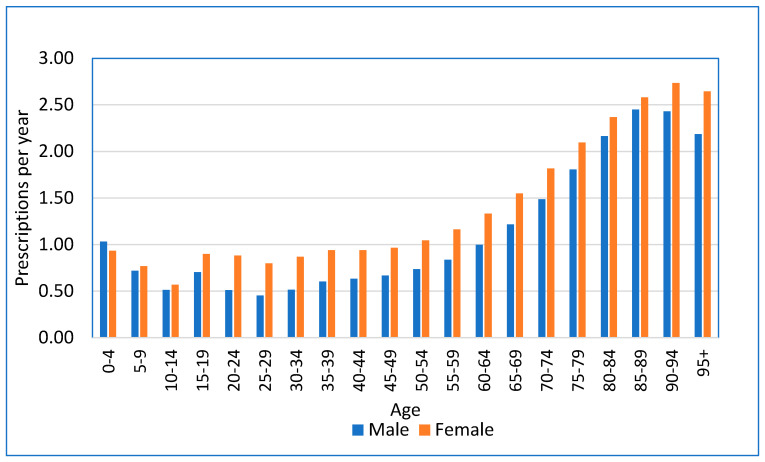
Antimicrobial Prescriptions per Capita of Resident Population per Year by Age: Average 2013–2024.

**Figure 2:**
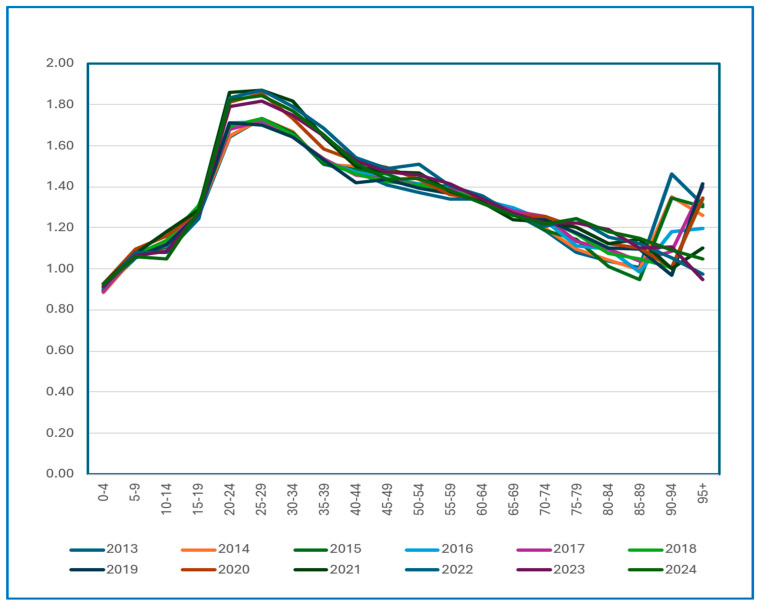
Female-to-male community antimicrobial dispensing ratio by age band and calendar year 2013–2024.

**Figure 3:**
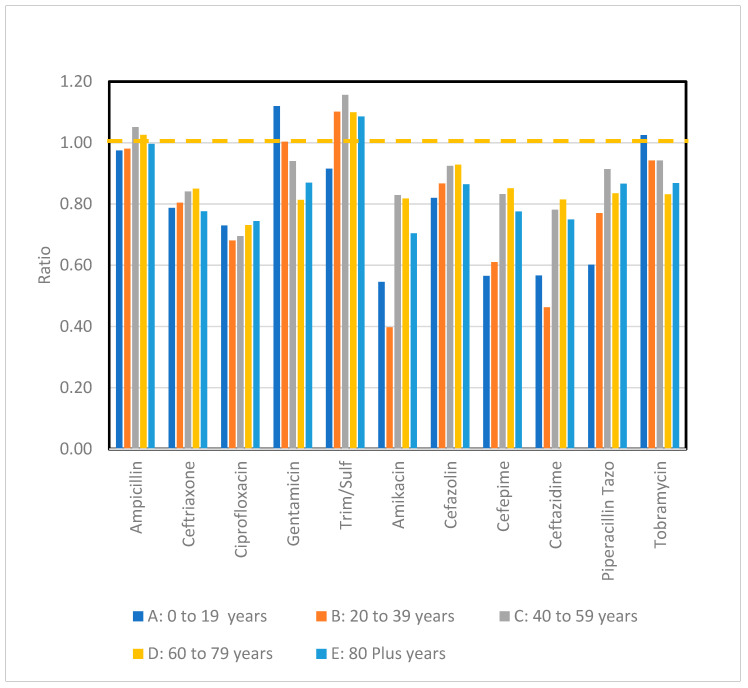
Female-to-male AMR ratio by antimicrobial and age band (less than 1 denotes lower resistance in women).

**Figure 4:**
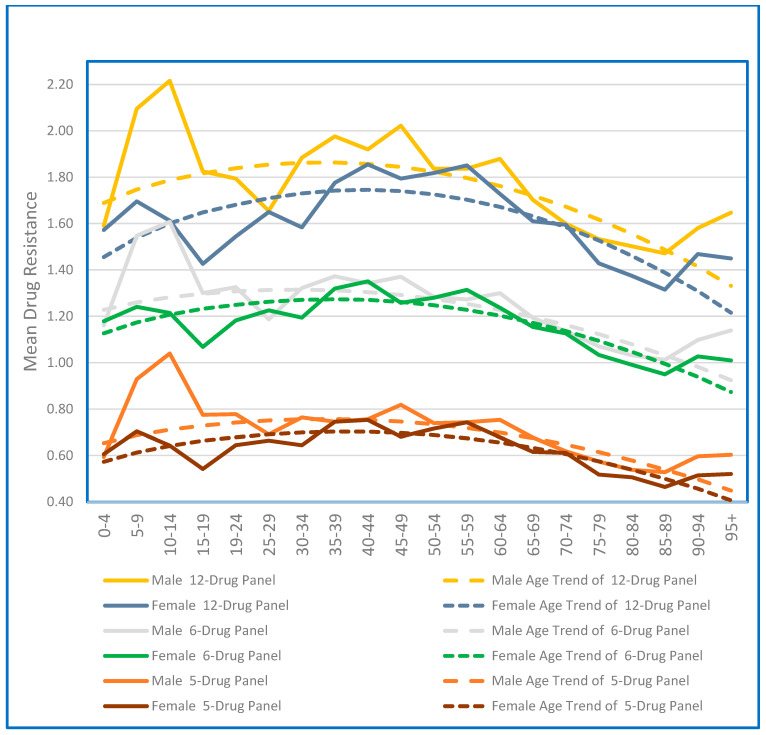
Mean drug resistance score by age band.

**Figure 5:**
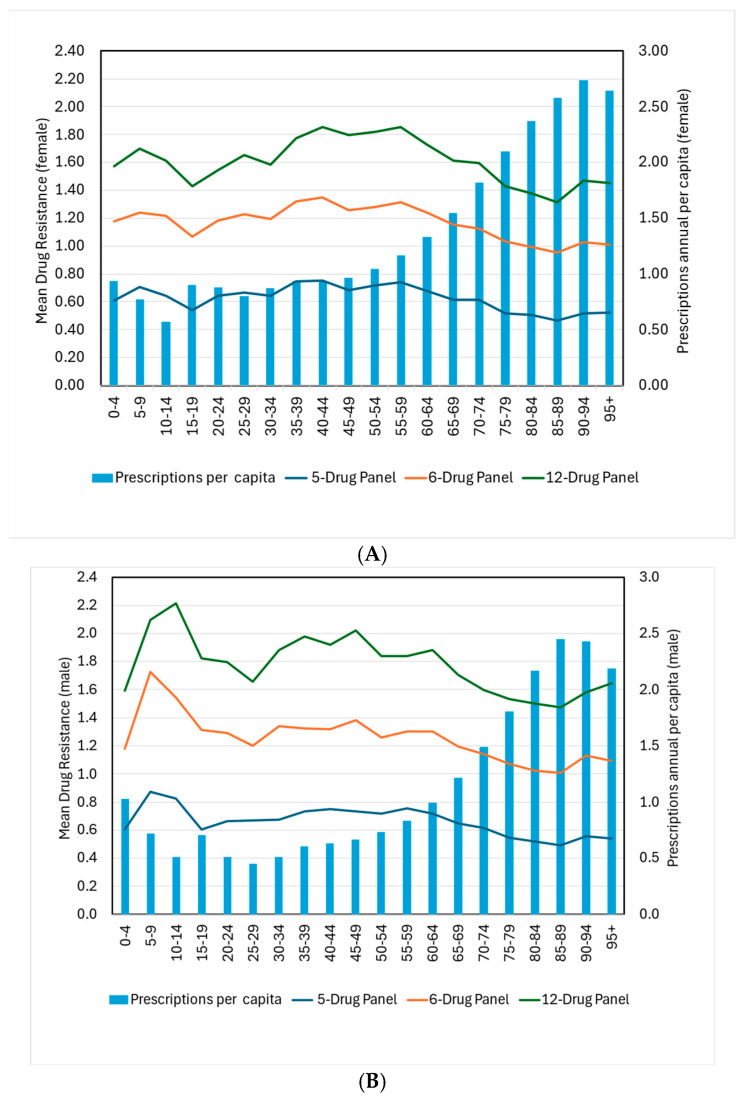
(**A**) Female mean drug resistance in 5-, 6- and 12-drug panels overlaid with antimicrobial dispensing by age band. (**B**) Male mean drug resistance in 5-, 6- and 12-drug panels overlaid with antimicrobial dispensing by age band. Legend: The left-hand y axis denotes the mean antibiotic resistance level for the drug panel. The right-hand y axis, the annual prescription rate per capita for that age group.

**Figure 6:**
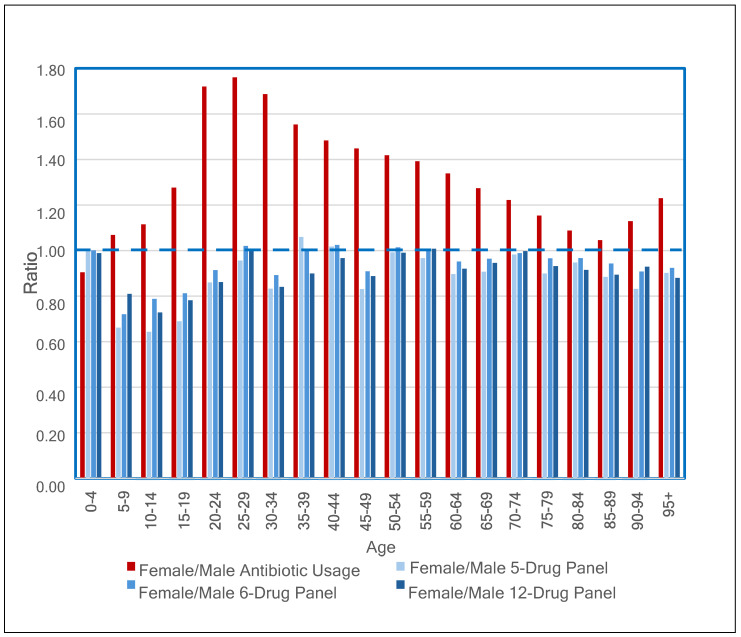
Female-to-male use ratio versus female-to-male resistance ratio by age (less than 1 denotes lower resistance or usage in women).

Across 44,792 episodes of community-onset *E. coli* bloodstream infection, antimicrobial use and antimicrobial resistance exhibited markedly discordant patterns across age and sex. Antimicrobial prescribing was substantially higher in females than males and increased progressively with age, with individuals aged ≥80 years receiving approximately three times more antimicrobials than those aged 25–30 years (Figure 1, Figure 2 and Figure 3). In contrast, antimicrobial resistance did not follow this pattern (Table 1 and Table 2 and Figure 4, Figure 5 and Figure 6). Resistance was consistently lower in females despite higher antimicrobial use and, after peaking in early adulthood, declined with increasing age despite substantial increases in exposure. This inverse relationship between antimicrobial use and resistance was observed across antimicrobial classes, composite resistance measures, calendar years, and jurisdictions.

From 2013 to 2024 females were dispensed approximately 23% more antimicrobials under the national PBS than males. The age-related per capita pattern of females using more antimicrobials than males was stable over the past decade. Data on the relative female to male usage is provided by 5-year age bands for each calendar year (Figure 2). Detailed tabular data are available in Supplementary Tables S3 and S4. For those aged 90–94 years during calendar years 2013 to 2016, and for 95+ persons during calendar years 2013 to 2020, there was an increase in the use of antimicrobials in males relative to females; however, the population size of these age groups is smaller and more liable to episodic volatility.

### 3.2. Antimicrobial Resistance and Sex

Antimicrobial resistance was generally, though not always, lower in females in comparison to males. Figure 3 summarizes by age and sex the ratio of antimicrobial resistance rates for individual drugs in the 12-Drug panel excluding unreliable small sample data on meropenem (refer Table 1). There are 40,587 episodes in the 12-drug panel and 44,792 episodes in the 5-drug and 6-drug panels. For the 12-drug panel cefazolin results were not available for 4205 episodes. Antimicrobial resistance rates by sex, age category for 12 drugs are summarised in Supplementary Table S5.

**Table 1:** All Ages Female to Male *E. coli* Resistance Odds Ratio.

Drug	Female/Male Resistance Odds Ratio	Lower Confidence Interval	Upper Confidence Interval
Ampicillin	1.05	1.01	1.09
Ceftriaxone	0.81	0.77	0.86
Ciprofloxacin	0.70	0.66	0.74
Gentamicin	0.88	0.82	0.94
Meropenem	1.03	0.45	2.35
Trimethoprim sulfamethoxazole	1.16	1.12	1.21
Amikacin	0.75	0.63	0.89
Cefazolin	0.88	0.84	0.92
Cefepime	0.79	0.71	0.87
Ceftazidime	0.74	0.68	0.80
Piperacillin-tazobactam	0.83	0.77	0.90
Tobramycin	0.88	0.83	0.94

In nearly all age groups, antimicrobial resistance rates were higher for male patients than for female patients for cefazolin, ciprofloxacin, aminoglycosides, cefepime, ceftazidime, ceftriaxone, and for average resistance across multidrug panels. For ampicillin and trimethoprim/sulfamethoxazole resistance for females in most age groups was higher than for males. Taken over the entire sample, Table 1 shows the drug-specific relative difference between male and female resistance rates as odds ratios, along with 95% confidence intervals. All differences were statistically significant except for meropenem where the number of resistant isolates was too small to reliably assess sex differences for that single drug.

Mean drug resistance is shown in Table 2 by sex for each panel. To illustrate interpretation, using the 12-drug panel for female patients, the average infection is resistant to 1.57 drugs, whilst for male patients, it is resistant to 1.66 drugs. In all three panels, average drug resistance is higher in male patients than in female patients, and the differences are statistically significant (*p* < 0.01).

**Table 2:** Mean Drug Resistance by Sex in Each Drug Panel.

Antimicrobial Panel	Female	Male	Difference	*p*-Value
5-Drug Panel	0.60	0.64	–0.04	<0.001
6-Drug Panel	1.13	1.16	–0.03	0.007
12-Drug Panel	1.57	1.66	–0.09	<0.001

### 3.3. Mean Drug Resistance, Age and Sex

The age-related pattern of mean drug resistance is shown in Figure 4. Antimicrobial resistance rates between females and males varied over individual years, but overall mean drug resistance was lower in females than in males.

Mean drug resistance tended to be higher in isolates from children; however, there were relatively few data points in the age range from 5 to 15 years (216 observations in the full sample). The overall pattern showed average drug resistance rising until ages between 30 and 40 years and then falling. A quadratic equation in age and age squared was fitted by ordinary least squares to each of the mean drug resistance curves in each panel in Figure 4 and the results are overlaid as dotted lines. The regressions were fitted to the underlying unit data of 44,792 episodes for the 5- and 6-drug panels, and 40,587 episodes for the 12-drug panel. In all cases the estimated coefficients in both age and age squared were statistically significant. Regression coefficients, *p*-values and associated statistics are detailed in Supplementary Table S6 for each drug panel. Regression statistics are given in Supplementary Table S7. Depending on the drug panel, the regression equations showed estimated peak age for antimicrobial resistance in females varied from age 36.8 to 41.4 years, and from age 31.2 to 35.4 years in males. The estimated age at which mean drug resistance peaks are summarised in Supplementary Table S8. In each drug panel, mean drug resistance is estimated to peak at a slightly older age for females.

### 3.4. Antimicrobial Resistance, Age and Antimicrobial Usage

Figure 5A,B overlay age-specific antimicrobial usage against mean drug resistance for female patients and male patients, respectively. From ages 30–40 to late age, antimicrobial usage rises in the order of three times, while mean antimicrobial resistance falls approximately 20%. The pattern is similar for both sexes.

### 3.5. Antimicrobial Resistance, Sex, and Antimicrobial Usage by Age Versus Comparative Resistance

Although the ages between 20 and 45 years are not a life stage of high absolute antimicrobial usage, usage in females was 40% to 70% higher than in males. Despite this antimicrobial usage pattern, antimicrobial resistance remained generally lower for females. In only 4 of the 15 ratios of average drug resistance in Figure 6 for ages 20 to 45 years, did males have higher antimicrobial resistance.

Through calendar time, male mean drug resistance rates remained above those for females (see Supplementary Table S9). For all three drug panels and for each year 2013–2024, average drug resistance was higher in males except for the 6-drug panels in 2016–2018 and 2023 where antimicrobial resistance scores were about 2% higher in females. However, antimicrobial usage remained consistently more than 20% higher in females compared to males over each year of the study. The ratio of female to male patient mean antimicrobial resistance for 5-, 6- and 12-drug panels and the ratio of females to males’ per capita antimicrobial usage by calendar year 2013–2024 is summarised in Supplementary Table S9.

The Northern Territory was the only jurisdiction in which mean resistance was higher in females. Because the Northern Territory accounts for a small proportion of the Australian population and has distinctive demographic, geographic, healthcare access, and burden-of-disease characteristics, this finding should be interpreted cautiously and warrants separate focused analysis. The female and male patient average drug resistance by jurisdiction is summarised in Supplementary Table S10.

## 4. Discussion

In this large national study of community-onset *E. coli* bloodstream infections, we identified a consistent and counterintuitive demographic pattern: groups with higher antimicrobial use—particularly females and older adults—did not exhibit higher antimicrobial resistance at the population level. Instead, resistance was generally lower in females than in males and declined with increasing age after early adulthood, despite substantial increases in antimicrobial exposure. These patterns were observed across antimicrobial classes, composite resistance measures, calendar years, and jurisdictions.

These findings are not readily explained by a simple model in which antimicrobial consumption is the dominant determinant of resistance at the population level. Under such a model, resistance would be expected to increase with greater exposure. In contrast, we observed an inverse relationship across key demographic strata. While this does not negate the well-established role of antimicrobial use in selecting for resistance within individuals, it suggests that additional mechanisms are required to explain how resistance is distributed across populations.

The comparison is ecological. Antimicrobial dispensing was measured in the general community population, whereas resistance was measured among patients who developed community-onset bloodstream infection. These populations are related but not identical. Therefore, the analysis cannot determine whether individuals with resistant infections had greater or lesser prior antimicrobial exposure. The value of the comparison is instead to test whether broad population-level demographic gradients in prescribing are mirrored by comparable gradients in resistance.

A more consistent interpretation and our hypothesis is that population-level resistance patterns reflect a combination of within-host selection and between-host transmission. This interpretation remains hypothetical because the present study did not directly measure colonisation, transmission events, infection source, travel, environmental exposure, prior healthcare contact, or individual antimicrobial histories. In this framework, antimicrobial use contributes to the emergence and amplification of resistance within individuals, but the prevalence of resistance in clinical infections depends substantially on exposure to resistant organisms circulating in the community, healthcare settings, and the environment. For *E. coli*, such exposure pathways include food systems, water and sanitation infrastructure, environmental contamination, travel, and person-to-person transmission and demonstrate why a One Health approach is so important [6,7,8,9,10,11,12,13]. Variation in these exposures across age and sex could contribute to the observed demographic differences in resistance.

The age-related pattern is particularly informative. Resistance peaked in early adulthood and declined thereafter, despite a marked increase in antimicrobial use with age. This pattern is difficult to reconcile with a monotonic consumption–resistance relationship and instead suggests that exposure and transmission may vary across the life course. For example, differences in occupational exposure, travel, childcare contact, or healthcare interactions may influence the probability of acquiring resistant organisms at different ages.

The consistency of the aggregated usage pattern provides confidence that the aggregation of data over the 12-year data period provides reliable insight into the age and sex pattern of national antimicrobial usage. Although the ages between 20 and 45 years are not a life stage of high absolute antimicrobial usage, usage in females was 40% to 70% higher than in males. Despite this antimicrobial usage pattern, antimicrobial resistance remained generally lower for females. In only 4 of the 15 ratios of average drug resistance in Figure 6 for ages 20 to 45 years, did females have higher antimicrobial resistance.

Similarly, the consistently lower resistance observed in females, despite higher antimicrobial use, suggests that factors other than prescribing volume may influence resistance risk. Differences in infection source are likely to be relevant. *E. coli* bloodstream infections in females are more commonly associated with urinary tract infections, whereas males more often present with more complex or healthcare-associated infections, which may be enriched for resistant organisms. Differences in healthcare exposure, comorbidities, or prior hospitalisation may also contribute. Behavioural and environmental factors, including hygiene practices, and patterns of exposure to resistant organisms, may further influence acquisition risk [30]. These factors may result in lower acquiescence rates in some groups and so lower carriage of antimicrobial resistant bacteria. While our data does not allow direct assessment of these mechanisms, they provide plausible explanations for the observed dissociation between antimicrobial use and resistance. But this does not explain the increase in the absolute numbers of infections which occurs with increasing age in both sexes [31], but with an opposite effect being seen in antimicrobial resistance rates in people as they become older, despite increased antimicrobial use.

A recent global study from Finland [16] on human gut metagenomes, reported that in “richer” countries more antimicrobial resistant genes were carried by females, but the opposite was however seen in poorer countries. A recent large-scale metagenomic study also examined the relationship between population-level antibiotic consumption and antimicrobial resistance in the human gut microbiome across multiple countries [32]. That study demonstrated a strong association between national antibiotic use and the abundance and diversity of antimicrobial resistance genes in commensal gut bacteria, largely driven by mobile resistance elements shared across species. However, this association was much weaker and inconsistent for pathogenic bacteria, and substantial heterogeneity between countries was observed, with China representing a notable outlier. These findings suggest that population-level antibiotic use may strongly shape the background resistomes within the human microbiome, without necessarily translating into higher resistance rates in invasive bacterial pathogens. Our findings in community-onset *E. coli* bloodstream infections are consistent with this interpretation and support the view that antimicrobial consumption volume alone is insufficient to explain resistance patterns in clinically significant infections.

Transmission dynamics operating through socioeconomic exposure pathways [7,8,9,10], including sanitation, water, health system infrastructure, housing and population-level mixing, are therefore likely to be very important determinants of resistance in invasive infections. This distinction underscores the importance of considering pathogen-specific transmission and acquisition pathways when interpreting population-level antimicrobial resistance data.

These results are consistent with a growing body of literature indicating that antimicrobial resistance correlates not only with antimicrobial consumption but also with broader ecological and socio-environmental factors, including sanitation, infrastructure, and governance [7,8,9,10]. They may also help explain why reductions in antimicrobial use, although essential, have not always led to proportional reductions in resistance at the population level. Together, these observations support a more integrated model in which antimicrobial use is one component of a wider system influencing resistance dynamics.

This study has implications for surveillance and policy. Current surveillance systems, including GLASS, largely report antimicrobial use and resistance at aggregated national levels and do not routinely disaggregate by age and sex [25]. Our findings demonstrate that important and potentially informative patterns may be obscured by such aggregation. Routine stratification by demographic variables could improve interpretation of surveillance data and help identify underlying drivers of resistance.

The findings also have implications for both intervention strategies and for future research. Efforts to optimise antimicrobial use remain critical and are strongly supported by evidence at the individual level. However, our results suggest that interventions focusing solely on reducing antimicrobial consumption may be insufficient to address population-level resistance patterns in pathogens such as *E. coli*. Greater attention to between-host transmission pathways—including infection prevention, sanitation, food safety, and environmental controls—are required to complement stewardship efforts.

## 5. Limitations

This study has several important limitations. First, the analysis is ecological and does not link individual antimicrobial exposure to resistance outcomes. As such, causal inferences cannot be made, and the findings should be interpreted as hypothesis-generating. While the data sources differ, both data sources are representative of the national populations and are used for Australia’s national antimicrobial use and resistance surveillance data. The PBS data are national level data and thus representative of national antimicrobial use. The bloodstream infection data are derived for the national antimicrobial resistance surveillance program and are thus also representative of the national population resistance patterns. Second, antimicrobial use was measured using community dispensing data and does not capture inpatient prescribing, which may be particularly relevant for some patient groups. Third, we were unable to adjust for individual-level factors such as comorbidities, prior healthcare exposure, infection source, or recent hospitalisation, all of which may influence resistance risk and could differ systematically by age and sex. These are likely major determinants of resistance patterns and should not be treated as secondary limitations. For example, males with E. coli bacteraemia often present with more complicated urinary or healthcare-associated infections, which could independently explain higher resistance rates. Fourthly, laboratories collected “either all, or up to 200 isolates” annually. This raises concerns regarding sampling representativeness and possible site-level selection bias. Fifth, the decline in resistance among older age groups may reflect survivor bias, differences in blood culture practices, or changing infection syndromes by age. Lastly, the analysis was restricted to *E. coli* bloodstream infections and may not generalise to other pathogens or infection types.

Despite these limitations, the consistency of the observed patterns across multiple dimensions—including age, sex, antimicrobial class, and time—suggests that they are unlikely to be explained by random variation alone. However, further studies incorporating individual-level data, detailed exposure histories, and broader genomic surveillance are needed to better understand the mechanisms underlying these findings at the population level.

## 6. Conclusions

In a large national dataset of community-onset *E. coli* bloodstream infections, demographic patterns of antimicrobial resistance were not consistent with a simple consumption-driven model. Resistance was lower in groups with higher antimicrobial use and declined with age despite increasing exposure. The data support that antimicrobial consumption alone does not fully explain the observed demographic patterns, but they do not refute antimicrobial selection pressure as a major driver of AMR. These findings suggest that population-level resistance patterns are shaped by a combination of antimicrobial use and transmission-related factors. Addressing antimicrobial resistance will require strategies that integrate antimicrobial stewardship with measures targeting the spread and transmission of resistant organisms.

## Data Availability

The original contributions presented in this study are included in the article/Supplementary Materials. Further inquiries can be directed to the corresponding author.

## Supplementary Materials

The following supporting information can be downloaded at: https://www.mdpi.com/article/10.3390/pathogens15070670/s1, Table S1: Antimicrobials studied; Table S2: Sample Size by Age Category and Sex; Table S3: Antimicrobial Prescriptions per Capita of Resident Population per Year by Age Category and Sex 2013–2024; Table S4: Ratio of Female to Male Antimicrobial Prescriptions per Capita of Resident Population per Year by Age Category and Sex 2013–2024; Table S5: Antimicrobial Resistance Rates by Sex, Age Category for 12 Drugs; Table S6: Regression Coefficients and *p*-values for Multi-Drug Resistance Against Quadratic Function in Age; Table S7: Regression statistics; Table S8: Estimated Age at which Mean Drug Resistance Peaks; Table S9: Ratio of Female to Male Patient Mean Drug Resistance for 5-, 6- and 12-Drug Panels and the Ratio of Female to Male Patient per Capita Antimicrobial Usage by Calendar Year 2013–2024; Table S10: Female and Male Patient Mean Drug Resistance Rates by Jurisdiction; Table S11: Participating members of AGAR in 2024.
