# Supplementary material for: Population-Level Uncoupling of Antimicrobial Usage and Resistance in Community-Onset Escherichia coli Bloodstream Infections
Source: Pathogens. 2026 Jun 25;15(7):670. doi: 10.3390/pathogens15070670 (PMC13415096; doi:10.3390/pathogens15070670)
Supplement: Supplementary file 1 [file pathogens-15-00670-s001.zip › pathogens-4343506-supplementary.pdf]

Supplementary Information

Supplementary Table 1: Antimicrobials studied

Agent	Number	Included	12-drug	6-drug	5-drug
Amikacin	45,319	Yes	Yes	–	–
Amoxicillin-clavulanate acid (fixed)	4,994	–[a]	–	–	–
Ampicillin	45,300	Yes	Yes	Yes	–
Aztreonam	7,307	–[b]	–	–	–
Cefalexin	5,172	–[c]	–	–	–
Cefazolin	40,989	Yes	Yes	–	–
Cefepime	45,318	Yes	Yes	–	–
Ceftazidime	45,324	Yes	Yes	–	–
Ceftriaxone	45,329	Yes	Yes	Yes	Yes
Cefuroxime	2,843	–[b]	–	–	–
Ciprofloxacin	45,303	Yes	Yes	Yes	Yes
Ertapenem	7,198	–[b]	–	–	–
Fosfomycin	5,169	–[b]	–	–	–
Gentamicin	45,315	Yes	Yes	Yes	Yes
Imipenem	5,105	–[b]	–	–	–
Mecillinam	2,722	–[c]	–	–	–
Meropenem	45,316	Yes	Yes	Yes	Yes
Nitrofurantoin	43,122	–[c]	–	–	–
Norfloxacin	41,217	–[c]	–	–	–
Piperacillin-tazobactam	45,070	Yes	Yes	–	–
Ticarcillin-clavulanate	38,177	Yes	–	–	–
Tigecycline	5,068	–[b]	–	–	–
Tobramycin	45,214	Yes	Yes	–	–
Trimethoprim	43,021	–[c]	–	–	–
Trimethoprim-sulfamethoxazole	45,260	Yes	Yes	Yes	Yes

– Not included
a Insufficient numbers tested using the EUCAST fixed formulation
b insufficient numbers (<10% of total)
c breakpoints for uncomplicated urinary tract infection only

Supplementary Table 2 : Sample Size by Age Category and Sex

	**5-Drug & 6-Drug Panels**			**12-Drug Panel**		
**Age Category**	**Female**	**Male**	**Total**	**Female**	**Male**	**Total**
A: 0-4	459	699	1,158	416	635	1,051
B: 5-9	54	47	101	46	42	88
C: 10-14	56	59	115	49	51	100
D: 15-19	251	89	340	237	80	317
E: 20-24	577	140	717	520	126	646
F: 25-29	741	154	895	674	139	813
G: 30-34	804	248	1,052	713	224	937
H: 35-39	773	273	1,046	709	247	956
I: 40-44	818	377	1,195	761	348	1,109
J: 45-49	978	555	1,533	903	505	1,408
K: 50-54	1,269	818	2,087	1,155	747	1,902
l: 55-59	1,409	1,228	2,637	1,293	1,108	2,401
M: 60-64	1,707	1,692	3,399	1,548	1,536	3,084
N: 65-69	2,041	2,206	4,247	1,847	1,997	3,844
O: 70-74	2,410	2,489	4,899	2,188	2,257	4,445
P: 75-79	2,747	2,613	5,360	2,512	2,364	4,876
Q: 80-84	2,992	2,660	5,652	2,674	2,414	5,088
R: 85-89	2,720	2,121	4,841	2,439	1,918	4,357
S: 90-94	1,624	1,075	2,699	1,471	970	2,441
T: 95+	540	279	819	472	252	724
Total	24,970	19,822	44,792	22,627	17,960	40,587

Supplementary Table 3: Antimicrobial Prescriptions per Capita of Resident Population per Year by Age Category and Sex 2013-2024

	2013	2014	2015	2016	2017	2018	2019	2020	2021	2022	2023	2024	All
**Female**	1.67	1.68	1.68	1.54	1.51	1.44	1.43	1.10	1.07	1.17	1.16	1.28	1.39
0-4	1.30	1.30	1.23	1.02	0.95	0.90	0.88	0.51	0.71	0.82	0.78	0.80	0.93
10-14	0.73	0.73	0.78	0.68	0.64	0.55	0.56	0.36	0.36	0.43	0.44	0.56	0.57
15-19	1.10	1.11	1.12	1.04	0.99	0.92	0.90	0.69	0.69	0.76	0.70	0.76	0.90
20-24	1.05	1.08	1.08	1.00	0.94	0.89	0.88	0.70	0.73	0.81	0.70	0.73	0.88
25-29	0.96	1.00	1.00	0.91	0.85	0.81	0.80	0.62	0.63	0.70	0.63	0.66	0.80
30-34	1.07	1.09	1.08	1.00	0.94	0.89	0.87	0.65	0.67	0.74	0.69	0.74	0.87
35-39	1.12	1.15	1.15	1.09	1.03	0.96	0.96	0.70	0.71	0.81	0.78	0.82	0.94
40-44	1.13	1.14	1.14	1.06	1.01	0.97	0.96	0.73	0.70	0.81	0.78	0.85	0.94
45-49	1.13	1.18	1.19	1.11	1.07	1.00	0.99	0.75	0.70	0.81	0.78	0.86	0.96
50-54	1.23	1.26	1.26	1.19	1.15	1.09	1.09	0.83	0.77	0.88	0.84	0.92	1.04
55-59	1.41	1.43	1.44	1.34	1.29	1.23	1.21	0.88	0.84	0.94	0.93	1.01	1.16
5-9	1.03	1.03	1.06	0.88	0.82	0.71	0.73	0.43	0.49	0.64	0.65	0.74	0.77
60-64	1.64	1.68	1.65	1.54	1.48	1.41	1.39	1.00	0.95	1.07	1.05	1.13	1.33
65-69	1.92	1.95	1.95	1.75	1.69	1.64	1.63	1.19	1.11	1.22	1.21	1.32	1.55
70-74	2.20	2.23	2.22	2.11	2.07	1.96	1.91	1.44	1.30	1.41	1.41	1.53	1.82
75-79	2.48	2.51	2.52	2.26	2.26	2.25	2.22	1.72	1.63	1.74	1.72	1.84	2.09
80-84	2.70	2.73	2.74	2.59	2.57	2.52	2.52	1.99	1.86	1.98	2.04	2.19	2.37
85-89	2.71	2.83	2.85	2.70	2.81	2.78	2.76	2.26	2.20	2.25	2.29	2.52	2.58
90-94	2.97	2.93	3.06	2.72	2.81	2.78	2.92	2.50	2.35	2.45	2.50	2.83	2.73
95+	3.43	3.33	3.13	2.83	2.73	2.49	2.51	2.05	2.03	2.05	2.25	2.90	2.64
**Male**	1.34	1.36	1.37	1.26	1.22	1.18	1.17	0.88	0.87	0.95	0.95	1.05	1.13
0-4	1.44	1.47	1.37	1.15	1.07	0.99	0.95	0.55	0.76	0.89	0.85	0.86	1.03
10-14	0.66	0.66	0.70	0.61	0.57	0.48	0.50	0.31	0.30	0.40	0.41	0.53	0.51
15-19	0.88	0.87	0.88	0.82	0.76	0.70	0.71	0.54	0.54	0.61	0.55	0.59	0.70
20-24	0.64	0.65	0.65	0.58	0.56	0.52	0.51	0.38	0.39	0.44	0.39	0.40	0.51
25-29	0.56	0.58	0.58	0.53	0.49	0.47	0.47	0.33	0.34	0.37	0.35	0.36	0.45
30-34	0.65	0.66	0.65	0.61	0.57	0.54	0.53	0.37	0.37	0.41	0.40	0.42	0.51
35-39	0.74	0.76	0.76	0.71	0.67	0.63	0.63	0.44	0.43	0.48	0.47	0.50	0.60
40-44	0.76	0.76	0.77	0.72	0.69	0.66	0.68	0.48	0.47	0.52	0.51	0.56	0.63
45-49	0.81	0.82	0.81	0.77	0.74	0.70	0.69	0.50	0.48	0.54	0.53	0.60	0.67
50-54	0.89	0.90	0.90	0.84	0.82	0.78	0.78	0.58	0.53	0.58	0.58	0.64	0.73
55-59	1.00	1.02	1.02	0.96	0.93	0.89	0.88	0.64	0.61	0.67	0.66	0.73	0.84
5-9	0.97	0.97	1.00	0.82	0.77	0.67	0.69	0.39	0.45	0.59	0.61	0.70	0.72
60-64	1.23	1.25	1.23	1.15	1.10	1.05	1.05	0.75	0.72	0.79	0.79	0.85	1.00
65-69	1.51	1.51	1.51	1.35	1.32	1.30	1.29	0.94	0.90	0.96	0.95	1.04	1.22
70-74	1.85	1.86	1.87	1.71	1.64	1.58	1.54	1.15	1.05	1.17	1.15	1.26	1.49
75-79	2.29	2.29	2.20	2.03	2.00	1.92	1.88	1.43	1.36	1.40	1.40	1.47	1.81
80-84	2.60	2.61	2.71	2.36	2.35	2.35	2.29	1.77	1.65	1.71	1.71	1.85	2.16
85-89	2.70	2.84	3.00	2.73	2.71	2.65	2.51	2.05	1.93	2.00	2.07	2.19	2.45
90-94	2.03	2.17	2.28	2.30	2.59	2.76	3.02	2.49	2.35	2.32	2.26	2.59	2.43
95+	2.61	2.64	2.41	2.37	1.95	1.86	1.78	1.52	1.84	2.11	2.37	2.77	2.19

Supplementary Table 4: Ratio of Female to Male Antimicrobial Prescriptions per Capita of Resident Population per Year by Age Category and Sex 2013-2024

Age Category	2013	2014	2015	2016	2017	2018	2019	2020	2021	2022	2023	2024	All
0-4	0.90	0.89	0.90	0.89	0.89	0.91	0.92	0.93	0.93	0.91	0.92	0.93	0.91
5-9	1.06	1.06	1.06	1.08	1.07	1.07	1.06	1.09	1.08	1.08	1.06	1.06	1.07
10-14	1.10	1.10	1.11	1.11	1.12	1.14	1.12	1.16	1.18	1.08	1.09	1.05	1.11
15-19	1.26	1.28	1.27	1.27	1.30	1.31	1.26	1.28	1.29	1.25	1.27	1.28	1.28
20-24	1.64	1.65	1.68	1.71	1.68	1.70	1.71	1.81	1.86	1.84	1.79	1.82	1.73
25-29	1.73	1.73	1.73	1.72	1.72	1.73	1.70	1.85	1.87	1.87	1.82	1.85	1.77
30-34	1.66	1.67	1.66	1.65	1.64	1.65	1.64	1.73	1.82	1.79	1.75	1.77	1.69
35-39	1.51	1.51	1.51	1.52	1.54	1.53	1.53	1.58	1.64	1.68	1.65	1.66	1.56
40-44	1.47	1.50	1.48	1.47	1.46	1.46	1.42	1.52	1.50	1.54	1.53	1.52	1.49
45-49	1.41	1.44	1.46	1.44	1.44	1.43	1.43	1.49	1.47	1.49	1.47	1.44	1.45
50-54	1.37	1.40	1.40	1.41	1.41	1.41	1.40	1.43	1.47	1.51	1.46	1.44	1.42
55-59	1.34	1.40	1.40	1.39	1.39	1.38	1.37	1.36	1.38	1.41	1.42	1.38	1.39
60-64	1.34	1.34	1.34	1.33	1.34	1.34	1.33	1.34	1.32	1.35	1.34	1.32	1.34
65-69	1.27	1.29	1.28	1.30	1.28	1.26	1.27	1.26	1.24	1.27	1.27	1.26	1.27
70-74	1.19	1.20	1.19	1.23	1.26	1.25	1.24	1.26	1.23	1.21	1.23	1.22	1.22
75-79	1.08	1.10	1.14	1.11	1.13	1.17	1.18	1.20	1.20	1.24	1.22	1.25	1.16
80-84	1.04	1.04	1.01	1.09	1.10	1.07	1.10	1.12	1.13	1.15	1.19	1.18	1.09
85-89	1.01	1.00	0.95	0.99	1.04	1.05	1.10	1.10	1.14	1.12	1.10	1.15	1.05
90-94	1.46	1.35	1.34	1.18	1.09	1.01	0.97	1.00	1.00	1.05	1.10	1.09	1.13
95+	1.31	1.26	1.30	1.20	1.40	1.34	1.41	1.34	1.10	0.97	0.95	1.05	1.21

Supplementary Table 5: Antimicrobial Resistance Rates by Sex, Age Category for 12 Drugs

Gender	Drug	0 to 19 years	20 to 39 years	40 to 59 years	60 to 79 years	80 Plus years	All Ages
Female	Ampicillin	55.6%	55.7%	57.5%	52.9%	49.0%	52.9%
Female	Ceftriaxone	7.9%	11.1%	12.1%	10.5%	9.2%	10.4%
Female	Ciprofloxacin	10.4%	12.8%	13.3%	11.6%	10.2%	11.6%
Female	Gentamicin	9.3%	8.7%	10.2%	7.5%	6.5%	7.9%
Female	Meropenem	0.12%	0.07%	0.04%	0.08%	0.01%	0.05%
Female	Trimethoprim-sulfamethoxazole	31.8%	35.0%	36.7%	29.9%	23.5%	29.7%
Female	Amikacin	0.7%	0.8%	1.5%	1.0%	0.8%	1.0%
Female	Cefazolin	17.6%	20.4%	23.4%	20.6%	18.9%	20.5%
Female	Cefepime	1.7%	2.7%	3.4%	3.4%	3.0%	3.1%
Female	Ceftazidime	3.3%	4.1%	5.7%	5.3%	4.9%	5.0%
Female	Piperacillin tazobactam	3.9%	4.6%	6.7%	5.5%	5.0%	5.4%
Female	Tobramycin	9.5%	9.4%	11.4%	8.1%	6.9%	8.5%
Male	Ampicillin	57.0%	56.8%	54.7%	51.5%	49.2%	51.8%
Male	Ceftriaxone	10.1%	13.7%	14.4%	12.3%	11.9%	12.5%
Male	Ciprofloxacin	14.2%	18.8%	19.1%	15.9%	13.7%	15.8%
Male	Gentamicin	8.3%	8.7%	10.8%	9.3%	7.4%	8.9%
Male	Meropenem	0.0%	0.1%	0.0%	0.1%	0.1%	0.1%
Male	Trimethoprim-sulfamethoxazole	34.8%	31.8%	31.7%	27.2%	21.7%	26.7%
Male	Amikacin	1.3%	2.1%	1.8%	1.2%	1.2%	1.3%
Male	Cefazolin	21.5%	23.5%	25.4%	22.2%	21.9%	22.6%
Male	Cefepime	3.0%	4.4%	4.0%	3.9%	3.9%	3.9%
Male	Ceftazidime	5.8%	9.0%	7.4%	6.5%	6.5%	6.7%
Male	Piperacillin tazobactam	6.5%	6.0%	7.3%	6.6%	5.8%	6.4%
Male	Tobramycin	9.3%	9.9%	12.1%	9.7%	8.0%	9.5%

Supplementary Table 6: Regression Coefficients and p-values for Multi-Drug Resistance Against Quadratic Function in Age

	Intercept	Age	Age^2
12 Drug Female	1.44953	0.01431	-0.00017
	<0.0001	<0.0001	<0.0001
12 Drug Male	1.68604	0.01006	-0.00014
	<0.0001	<0.0001	<0.0001
6 Drug Female	1.12303	0.00818	-0.00011
	<0.0001	<0.0001	<0.0001
6 Drug Male	1.22619	0.00570	-0.00009
	<0.0001	0.00119	<0.0001
5 Drug Female	1.12303	0.00818	-0.00011
	<0.0001	<0.0001	<0.0001
5 Drug Male	0.65161	0.00591	-0.00008
	<0.0001	<0.0001	<0.0001

Supplementary Table 7: Regression statistics

12 Drug Female

Regression Statistics	
Multiple R	0.07149
R Square	0.00511
Adjusted R Square	0.00502
Standard Error	2.01686
Observations	22627

ANOVA

	df	*SS*	*MS*	*F*	*Significance F*
Regression	2	472.80610	236.40305	58.1167	0.00000
Residual	22624	92028.28702	4.06773		
Total	22626	92501.09312			

	Coefficients	*Standard Error*	*t Stat*	*P-value*	*Lower 95%*	*Upper 95%*	*Lower 95.0%*	*Upper 95.0%*
Intercept	1.44953	0.07278	19.91636	0.0000	1.30688	1.59219	1.30688	1.59219
Age	0.01431	0.00269	5.31151	0.0000	0.00903	0.01959	0.00903	0.01959
Age^2	-0.00017	0.00002	-7.30852	0.0000	-0.00022	-0.00013	-0.00022	-0.00013

6 Drug Female

Regression Statistics	
Multiple R	0.08639
R Square	0.00746
Adjusted R Square	0.00738
Standard Error	1.27291
Observations	22627

ANOVA

	df	*SS*	*MS*	*F*	*Significance F*
Regression	2	275.66752	137.83376	85.0666	0.00000
Residual	22624	36657.75538	1.62030		
Total	22626	36933.42290			

	Coefficients	*Standard Error*	*t Stat*	*P-value*	*Lower 95%*	*Upper 95%*	*Lower 95.0%*	*Upper 95.0%*
Intercept	1.12303	0.04593	24.44845	0.0000	1.03299	1.21306	1.03299	1.21306
Age	0.00818	0.00170	4.81208	0.0000	0.00485	0.01152	0.00485	0.01152

Age^2	-0.00011	0.00001	-7.44654	0.0000	-0.00014	-0.00008	-0.00014	-0.00008

5 Drug Female

Regression Statistics	
Multiple R	0.08462
R Square	0.00716
Adjusted R Square	0.00707
Standard Error	0.95733
Observations	22627

ANOVA

	df	*SS*	*MS*	*F*	*Significance F*
Regression	2	149.52710	74.76355	81.5763	0.00000
Residual	22624	20734.59275	0.91649		
Total	22626	20884.11986			

	Coefficients	*Standard Error*	*t Stat*	*P-value*	*Lower 95%*	*Upper 95%*	*Lower 95.0%*	*Upper 95.0%*
Intercept	0.57028	0.03455	16.50747	0.0000	0.50256	0.63799	0.50256	0.63799
Age	0.00685	0.00128	5.35634	0.0000	0.00434	0.00936	0.00434	0.00936
Age^2	-0.00009	0.00001	-7.85634	0.0000	-0.00011	-0.00007	-0.00011	-0.00007

12 Drug Male

Regression Statistics	
Multiple R	0.05958
R Square	0.00355
Adjusted R Square	0.00344
Standard Error	2.18454
Observations	17960

ANOVA

	df	*SS*	*MS*	*F*	*Significance F*
Regression	2.00000	305.26669	152.63335	31.9837	0.00000
Residual	17957	85694.69856	4.77222		
Total	17959	85999.96526			

	Coefficients	*Standard Error*	*t Stat*	*P-value*	*Lower 95%*	*Upper 95%*	*Lower 95.0%*	*Upper 95.0%*
Intercept	1.68604	0.07774	21.68830	0.0000	1.53367	1.83842	1.53367	1.83842
Age	0.01006	0.00284	3.54554	0.0004	0.00450	0.01563	0.00450	0.01563
Age^2	-0.00014	0.00003	-5.42407	0.0000	-0.00019	-0.00009	-0.00019	-0.00009

6 Drug Male

Regression Statistics	
Multiple R	0.07063
R Square	0.00499
Adjusted R Square	0.00488

Standard Error	1.35359
Observations	17960

ANOVA

	df	SS	MS	F	Significance F
Regression	2	164.93323	82.46661	45.0098	0.00000
Residual	17957	32900.68320	1.83219		
Total	17959	33065.61643			

	Coefficients	Standard Error	t Stat	P-value	Lower 95%	Upper 95%	Lower 95.0%	Upper 95.0%
Intercept	1.22619	0.04817	25.45593	0.0000	1.13177	1.32061	1.13177	1.32061
Age	0.00570	0.00176	3.24236	0.0012	0.00225	0.00915	0.00225	0.00915
Age^2	-0.00009	0.00002	-5.62621	0.0000	-0.00012	-0.00006	-0.00012	-0.00006

5 Drug Male

Regression Statistics	
Multiple R	0.07274
R Square	0.00529
Adjusted R Square	0.00518
Standard Error	1.03361
Observations	17960

ANOVA

	df	SS	MS	F	Significance F
Regression	2	102.05466	51.02733	47.7627	0.00000
Residual	17957	19184.36488	1.06835		
Total	17959	19286.41954			

	Coefficients	Standard Error	t Stat	P-value	Lower 95%	Upper 95%	Lower 95.0%	Upper 95.0%
Intercept	0.65161	0.03678	17.71539	0.0000	0.57952	0.72371	0.57952	0.72371
Age	0.00591	0.00134	4.40155	0.0000	0.00328	0.00854	0.00328	0.00854
Age^2	-0.00008	0.00001	-6.68467	0.0000	-0.00011	-0.00006	-0.00011	-0.00006

Supplementary Table 8: Estimated Age at which Mean Drug Resistance Peaks

	Female	Male
12-Drug panel	41.4	35.4
6-Drug Panel	36.8	31.2
5-Drug Panel	36.8	35.6

Supplementary Table 9: Ratio of Female to Male Patient Mean Drug Resistance for 5-, 6- and 12-Drug Panels and the Ratio of Female to Male Patient per Capita Antimicrobial Usage by Calendar Year 2013-2024

Year	5-Drug Panel	6-Drug Panel	12-Drug Panel	Antimicrobial Usage
2013	0.92	0.98	0.92	1.24
2014	0.87	0.95	0.91	1.23
2015	0.83	0.90	0.88	1.23
2016	1.00	1.02	0.97	1.23
2017	0.98	1.02	0.97	1.24
2018	0.99	1.02	0.99	1.22
2019	0.93	0.98	0.96	1.23
2020	0.88	0.92	0.93	1.25
2021	0.95	0.97	0.98	1.23
2022	0.93	0.96	0.91	1.23
2023	1.00	1.03	0.99	1.22
2024	0.89	0.93	0.92	1.23

Supplementary Table 10: Female and Male Patient Mean Drug Resistance Rates by Jurisdiction

	Average of MDR 5 drugs		Average of MDR 6 Drugs		Average of MDR 12 Drugs	
	Female	Male	Female	Male	Female	Male
Australian Capital Territory	0.53	0.66	1.04	1.18	1.45	1.65
New South Wales	0.62	0.68	1.14	1.20	1.58	1.71
Northern territory	0.94	0.85	1.63	1.45	2.26	2.05
Queensland	0.54	0.58	1.06	1.07	1.42	1.53
South Australia	0.49	0.54	0.96	1.01	1.28	1.39
Tasmania	0.34	0.39	0.77	0.82	0.99	1.13
Victoria	0.60	0.66	1.14	1.20	1.57	1.71
Western Australia	0.64	0.70	1.20	1.25	1.65	1.79
Total	0.60	0.64	1.13	1.16	1.54	1.64

Supplementary Table 11: Participating members of AGAR in 2024

Hospitals	AGAR members
Alfred Hospital, Vic	Adam Jenney and Jacqueline Williams
Alice Springs Hospital, NT	James McLeod
Austin Hospital, Vic	Marcel Leroi and Elizabeth Grabsch
Cairns Base Hospital, Qld	Enzo Binotto and Annika Klein
Canberra Hospital, ACT	Peter Collignon and Susan Bradbury
Children's Hospital Westmead, NSW	Annaleise Howard-Jones and Zorana McDavitt
Concord Hospital, NSW	Thomas Gottlieb and John Huynh
Dandenong Hospital, Vic	Tony Korman and Kathryn Cisera
Fiona Stanley Hospital, WA	Shakeel Mowlaboccus and Denise Daley
Flinders Medical Centre, SA	Kelly Papanaoum and Xiao Ming Chen
Gold Coast University Hospital, Qld	Petra Derrington and Cheryl Curtis
Gosford Hospital, NSW	Gabrielle O'Kane and Nola Hitchick
Greenslopes Private Hospital, Qld	Jennifer Robson and Marianne Allen
Joondalup Hospital, WA	Shalinie Perera and Gemma Groves
Launceston General Hospital, Tas	Pankaja Kalukottege and Brooke Woolley
Liverpool Hospital, NSW	Michael Maley and Helen Ziochos
Mater Private Hospital, Townsville, Qld	Jennifer Robson and Marianne Allen
Monash Children’s Hospital, Vic	Tony Korman and Despina Kotsanas
Monash Medical Centre, Vic	Tony Korman and Despina Kotsanas
Nepean Hospital, NSW	James Branley and Linda Douglass
North-west regional Hospitals, WA	Michael Leung and Jacinta Bowman
Perth Children’s Hospital, WA	Christopher Blyth and Jacinta Bowman
Prince Charles Hospital, Qld	Robert Horvath and Jessica Bostock
Prince of Wales Hospital, NSW	Monica Lahra and Peter Huntington
Princess Alexandra Hospital, Qld	Naomi Runnegar and Anna Jones
Queensland Children's Hospital, Qld	Clare Nourse and Jessie Bostock
Royal Adelaide Hospital, SA	Morgyn Warner and Kija Smith
Royal Brisbane and Women's Hospital, Qld	Michael Thomas and Jessica Bostock
Royal Darwin Hospital, NT	Rob Baird and Jann Hennessy
Royal Hobart Hospital, Tas	Louise Cooley and Belinda McEwan
Royal Melbourne Hospital, Vic	Katherine Bond and Rose Cotronei
Royal North Shore Hospital, NSW	Shelanah Fernando and Angela Wong
Royal Perth Hospital, WA	Owen Robinson and Geoffrey Coombs
Royal Prince Alfred Hospital, NSW	Sebastiaan van Hal and Frances Jenkins
Royal Women’s and Children’s Hospital, Vic	Andrew Daley and Gena Gonis
Sir Charles Gairdner Hospital, WA	Ronan Murray and Jacinta Bowman
St John of God Hospital, Murdoch, WA	Sudha Pottumarthy-Boddu and Alicia Robinson
St Vincent’s Hospital, Melbourne, Vic	Amy Crowe and Lisa Brenton
St Vincent’s Hospital, Sydney, NSW	David Lorenz
Sydney Children’s Hospital, NSW	Monica Lahra and Peter Huntington
Westmead Hospital, NSW	Jonathan Iredell and Jignasa Purani
Wollongong Hospital, NSW	Peter Newton and Melissa Hoddle
Women’s and Children’s Hospital, SA	Morgyn Warner and Kija Smith
